# Philadelphia Agent

**Published:** 1842-03

**Authors:** 


*Philadelphia Agent.*—Dr. Roper, of Philadelphia, has kindly consented to act as agent for this journal in that place, and is authorised to receive subscriptions, and will hereafter see that the subscribers there are supplied with it as soon as it is issued from the press. Heretofore, we have had much difficulty in getting a suitable person to act as agent in that place, and in consequence, have received complaints from subscribers there on account of not having received the journal regularly. We trust there will be no cause for this in future.

